# Interpretation of the mechanism of action of antituberculosis drug bedaquiline based on a novel two‐ion theory of energy coupling in ATP synthesis

**DOI:** 10.1002/btm2.10106

**Published:** 2018-09-29

**Authors:** Sunil Nath

**Affiliations:** ^1^ Department of Biochemical Engineering and Biotechnology Indian Institute of Technology Delhi Hauz Khas New Delhi India

**Keywords:** bactericidal mode of action, bedaquiline, bedaquiline as an H^+^/K^+^ exchanger, bioenergetics, central role of succinate translocation in the creation of Δψ, F_1_F_O_‐ATP synthase, fluorescence quenching, H^+^/K^+^ antiport, mycobacteria, *Mycobacterium tuberculosis*, Nath's torsional mechanism of energy transduction and ATP synthesis, Nath's two‐ion theory of biological energy coupling, oxidative phosphorylation (OXPHOS), uncouplers

## Abstract

Tuberculosis (TB) claims the lives of 1.3 million people each year, more than any other bacterial infection. Hence great interest was generated in health communities upon the recent introduction of the new diarylquinoline anti‐TB drug, bedaquiline. Bedaquiline acts by binding to the c‐subunit in the membrane‐bound F_O_ portion of the F_1_F_O_‐adenosine triphosphate (ATP) synthase, the universal enzyme that produces the ATP needed by cells. However, the mechanism of killing by bedaquiline is not fully understood. Recent observations related to the bactericidal effects of bedaquiline, which show that it is a potent uncoupler of respiration‐driven ATP synthesis in *Mycobacterium smegmatis* are summarized. These observations are then interpreted from the standpoint of Nath's two‐ion theory of energy coupling in ATP synthesis (Nath, *Biophys. Chem*. 2017; 230:45–52). Especial importance is given to the interpretation of biochemical fluorescence quenching data, and the differences between the uncoupling induced by bedaquiline from that by the classical anionic uncouplers of oxidative phosphorylation are highlighted. Suggestions for new experiments that could lead to a better understanding of the uncoupling mechanism are made. A model of uncoupling action by the drug is presented, and the biochemical basis underlying uncoupling of ATP synthesis and lethality in mycobacteria is elucidated. The major biological implications arising from these novel insights are discussed. It is hoped that the analysis will lead to a more fundamental understanding of biological energy coupling, uncoupling and transduction, and to an integrated view for the design of novel antimicrobials by future research in the field.

## 1 INTRODUCTION

Developing countries are faced with their own peculiar afflictions and diseases, such as human tuberculosis (TB), that also provide opportunities for new drug development. If these developments could be connected with the author's fundamental research, especially on Nath's torsional molecular mechanism of the synthesis of the universal biological currency, adenosine triphosphate (ATP) that turns 20 years in 2018–2019,^1^ it would offer an appropriate topic for the Special Issue. Moreover, this topic had the approval of the Editor‐in‐Chief through his letter of invitation. It would be a fitting tribute to the pioneering and multifarious contributions of Bob Langer to drug development and pharmaceutical sciences in a life‐time of research.

**Figure 1:**
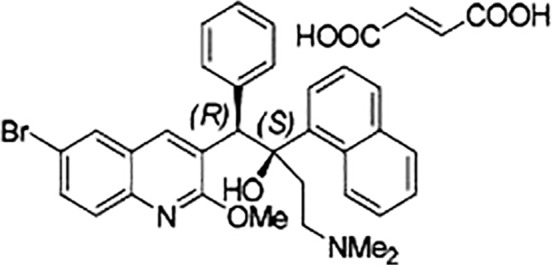
The chemical structure of the diarylquinoline drug, bedaquiline fumarate, generally known as bedaquiline, and marketed as SIRTURO. The first stereo‐label refers to the carbon atom with the phenyl group, while the second stereo‐label refers to the carbon atom harboring the hydroxyl group

TB continues to cause more loss of lives, 1.3 million people every year,^2^, ^3^ than any other bacterial infection. This makes the development of new antibiotics, especially against multidrug‐resistant (MDR) *Mycobacterium tuberculosis* urgent and important.^4^, ^5^, ^6^ The last drug for treating TB, rifampin, was introduced in 1971. Hence, great interest was generated when controlled clinical phase 2 trials revealed a potent bactericidal effect of the novel anti‐MDR TB drug, bedaquiline fumarate (generally called bedaquiline, and marketed as Sirturo) accompanied by a considerably shortened treatment time.^7^ The drug was granted accelerated Food and Drug Administration Approval.^8^ Bedaquiline is a member of the chemical class of diarylquinolines (Figure 1) and has been shown to exert its effects by binding to the c‐subunit in the membrane‐bound F_O_ portion of the F_1_F_O_‐ATP synthase.^9^, ^10^ The F_1_F_O_‐ATP synthase is the universal enzyme in animal mitochondria, plant chloroplasts and bacteria that produces the ATP needed by the cell to fuel its catabolic and anabolic reactions^11^, ^12^, ^13^ via the central process of oxidative phosphorylation (OXPHOS) or photophosphorylation.^14^, ^15^, ^16^

The F_1_F_O_‐ATP synthase has been shown to be essential for growth in *Mycobacterium smegmatis*.^17^ Furthermore, the mycobacteria catalyze ATP synthesis solely and do not operate in the reverse ATP hydrolysis mode. The mycobacterial ATP synthase has unique properties and selectivity to bedaquiline compared to its eukaryotic homolog.^18^ Detailed knowledge of the function of the F_1_F_O_‐ATP synthase is therefore central to a complete understanding of how targeting of the ATP synthase due to bedaquiline challenge leads to lethality of mycobacteria and for determination of the mode of action of bedaquiline.

## 2 OBSERVATIONS RELATED TO DELAYED EFFECTS OF BEDAQUILINE AND CELLULAR ATP DEPLETION

Bedaquiline shows in vitro activity and inhibits the growth of both drug‐sensitive and drug‐resistant *M. tuberculosis* strains and also in vivo activity against mycobacteria in TB patients. On treatment of growing or non‐growing mycobacterial cells with bedaquiline, time‐dependent killing has been observed.^9^ However, the mechanism of killing is unclear, but it does not involve collapse of the membrane potential, Δψ.^19^ A dose‐dependent decrease of intracellular ATP has been observed on treatment of *M. tuberculosis* cells with bedaquiline and this ATP depletion may be a determinant of the observed delayed onset of killing.^19^ However, mycobacterial cells can be depleted of ATP via deenergization methods, yet their viability is not compromised^20^ implying that cell death is not fully explained by ATP depletion. In fact, novel mechanisms alternative to ATP depletion are necessary to explain the potent bactericidal effects of bedaquiline.

## 3 RECENT OBSERVATIONS RELATED TO THE POTENT BACTERICIDAL ACTION OF BEDAQUILINE

Recently, it was shown that bedaquiline is a potent uncoupler of redox‐driven ATP synthesis in *M. smegmatis*.^21^ Structural analysis revealed that bedaquiline does not function as a general conductor of protons from one bulk aqueous phase to another; rather, it exerts its effects by binding to the c‐subunit in the F_O_ portion of ATP synthase.^10^ Classical uncouplers of OXPHOS dissipate both the ΔpH and the Δψ which are then recreated by respiratory activity leading to a futile cycle of ion transport uncoupled from ATP synthesis.^13^, ^22^ Uncoupling activity induced by the dinitrophenols has been quantitatively modeled using the principles of thermodynamics and transport phenomena by Jou and colleagues.^23^, ^24^ Such uncoupling is not lethal for *Escherichia coli*. However, for reasons that are not well understood, the uncoupling arising from bedaquiline challenge helps cause lethality in mycobacteria.

## 4 NOVEL TWO‐ION THEORY OF ENERGY COUPLING IN ATP SYNTHESIS

According to Mitchell's chemiosmotic theory,^25^ a delocalized electrical potential across bulk aqueous phases is an essential intermediate that is communicated far away and its energy used by the F_O_‐nanomotor to synthesize ATP by retro proton translocation. However, physiological ATP synthesis was demonstrated in the 1990s with single molecules of the ATP synthase purified and reconstituted into liposomes that contained no redox/photosystem complexes. Therefore, either no electrical potential is created,^26^ or a local potential, Δψ is created at the a–c interface in the membrane‐bound F_O_ portion of ATP synthase.^12^, ^13^, ^27^

A more detailed alternative Nath's torsional mechanism of ATP synthesis and two‐ion theory of energy coupling went beyond Mitchell's chemiosmotic theory.^13^ The theory was quantitative and contained mathematical equations that described the overall driving force of ATP synthesis arising from discrete proton and anion/countercation translocations in the membrane‐bound F_O_ portion of F_1_F_O_‐ATP synthase.^28^ A 10‐year experimental search^22^ established the identity of the elusive anion involved in energy transduction and ATP synthesis as a C4 dicarboxylic acid anion—succinate in animal mitochondria and malate in plant chloroplasts—although in certain bacteria a countertransport of Na^+^ or K^+^ was also proposed to be involved.^14^, ^16^, ^29^, ^30^, ^31^, ^32^, ^33^, ^34^, ^35^

The recent important experimental observations^21^ and the availability of a novel two‐ion theory of energy coupling in ATP synthesis^28^ provides further mechanistic insights into the bactericidal mode of action of bedaquiline, and helps in explanation and interpretation of the antibiotic's bactericidal effects. This ought to also lead to a more holistic view of biological energy coupling and transduction for the design of novel antimicrobials by future research in the field.

## 5 CHARACTERISTICS OF UNCOUPLING OF ATP SYNTHESIS IN *M. SMEGMATIS* BY BEDAQUILINE

Hards et al. showed that bedaquiline is a potent uncoupler of respiration‐driven ATP synthesis.^21^ They found that treatment of *M. smegmatis* cells with bedaquiline led to a 2.3‐fold increase in oxygen consumption, similar to that found with the classical anionic inhibitors of OXPHOS. This uncoupling effect was inferred to require binding of bedaquiline to the c‐subunit of the F_1_F_O_‐ATP synthase because a bedaquiline‐resistant isolate with a D32V point mutation in the c‐subunit that does not bind to the c‐oligomer did not show any stimulation of respiration. Calculation of the membrane potential, Δψ in whole cells of *M. smegmatis* by measurement of the distribution of the cationic probe [^3^H]TPP^+^ showed that 2 mg/L of bedaquiline did not affect the Δψ value of about 150 mV for up to 10 min after treatment with bedaquiline. The characteristics of the uncoupling behavior of bedaquiline were investigated further by fluorescence quenching of dyes in inverted membrane vesicles (IMVs) prepared by cell fractionation.

The establishment of a ΔpH in IMVs of *M. smegmatis* was measured by the quenching of fluorescence of the fluorescent probe acridine orange. Initiation of the reaction began upon incubation of IMVs with 5 mM succinate and led to quenching of the acridine orange fluorescence. The quenching reaction was complete and the fluorescence reached a low steady‐state value within 4 min. Addition of 50 μM carbonylcyanide m‐chlorophenylhydrazone (CCCP) uncoupler after this time led to reversal of the fluorescence quenching by capture of protons by the uncoupler and collapse of the ΔpH within 1 min. Similar reversal of quenching was observed upon administration of bedaquiline which was dose dependent, with complete reversal occurring at 8 mg/L of bedaquiline within a minute in wild‐type membranes. This reversal of fluorescence quenching was completely abolished in IMVs of the D32V mutant upon treatment with 1–8 mg/L bedaquiline concentration. However, 50 μM CCCP yielded complete recovery of the fluorescence of acridine orange within 1 min, as in wild‐type membranes.^21^

The presence of Δψ was measured by quenching of fluorescence of the dye Oxonol VI, and the reaction was initiated by incubation with 10 mM succinate. The quenching reaction was complete within 1 min in both wild‐type and D32V mutant membranes. Treatment with 4 and 8 mg/L bedaquiline did not lead to reversal of the fluorescence of Oxonol VI in wild‐type IMVs. However, incubation with 2 μM valinomycin led to almost complete reversal of the fluorescence within 30 s in wild‐type membranes. In the mutant, treatment with 4 or 8 mg/L of bedaquiline or incubation with 2 μM valinomycin caused no significant reversal of the fluorescence quenching.^21^

## 6 INTERPRETATION OF THE OBSERVATIONS ON UNCOUPLING ACTION OF BEDAQUILINE BASED ON NATH'S TWO‐ION THEORY OF ENERGY COUPLING

Incubation of IMVs of *M. smegmatis* with 5 mM succinate led to coupled proton translocation and progressive quenching of the acridine orange fluorescence by continual capture and sequestering of the protons by the fluorescent probe over time. Once the capacity of the fluorescent probe for H^+^ was satiated (typically after approximately 4 min), a low value of steady‐state fluorescence was achieved, which was a measure of the creation of ΔpH. As, according to this theory, there can be no continued translocation of H^+^ ions without the *coupled* cotransport of anions such as succinate or countertransport of cations such as K^+^, the transport of protons from inside to outside in the experiments must have been accompanied by a stoichiometric transport of succinate monoanion from inside to outside or of K^+^ ions from outside to inside through the aqueous access channels at the a–c interface of the F_O_ portion of ATP synthase.^27^, ^28^ A small contribution is possible from the neutral undissociated form of succinate that enters the aqueous access channels in F_O_ and yields a succinate monoanion and a proton at their respective binding sites in F_O_. However, our experimental data on the kinetically pure competitive inhibition found between uncoupler anions of three classes and succinate anions suggest the former explanation.^22^

That the uncoupler enters and binds as an anion we derive from the fact that various uncoupler anions compete with substrate anions like succinate for entry into mitochondria. Such competition phenomena are readily accounted for by the two‐ion theory of energy coupling within Nath's torsional mechanism of energy transduction and ATP synthesis. Addition of 50 μM CCCP uncoupler after ~4 min led to entry and binding of uncoupler anion in competition with succinate anion to a site on the a‐ or c‐subunit and to increasing reversal of the fluorescence quenching with time as progressive capture of protons by the CCCP anion took place in the vicinity of the binding sites due to the higher lipid solubility of the uncoupler (compared to physiological succinate anion), leading to formation of the uncharged form of the uncoupler and collapse of the ΔpH within 60 s. The uncharged species diffused away into the inside bulk aqueous phase, dissociated in that aqueous environment into uncoupler anion and proton, both of which were then pumped back by respiratory activity to restore the uncoupler gradient. The diffusion inwards of the neutral, uncharged species of uncoupler is very fast, compared to translocation separately of the individual ions and the slow conformational changes in the c‐subunit that these ion‐protein interactions induce.^12^ Hence the acceleration of cellular respiration that results in order to restore the ionic gradients by the redox side of OXPHOS, and how this phenotypic outcome arises originally from a drug–target interaction in F_O_ is also clarified.

The explanation for the dose‐dependent reversal of fluorescence quenching of acridine orange by bedaquiline is similar to that of CCCP uncoupler given above except that owing to its hydrophobic properties, bedaquiline enters and binds to its site on the c‐subunit in the membrane without competing for entry and binding with physiological succinate monoanions (or K^+^). This reversal of fluorescence quenching was completely abolished in IMVs of the D32V mutant upon bedaquiline treatment due to the inability of bedaquiline to bind to the mutant c‐subunit, thus impeding its ability to capture protons. As CCCP entry and binding to a different site on the a‐ or c‐subunit is not inhibited in the D32V mutant, 50 μM CCCP caused complete reversal of the fluorescence of acridine orange, exactly as in wild‐type membranes. It should, however, be noted that formation of the neutral form of the CCCP uncoupler also dissipates the Δψ created by uncoupler anion translocation and binding unlike during the uncoupling action of bedaquiline, which is a key difference and distinguishing feature of bedaquiline as compared to the classical uncouplers of OXPHOS.

We now turn to the data on quenching of Oxonol VI fluorescence and its reversal. The explanation for the rapid quenching of dye fluorescence is similar to that for acridine dyes except that the absorption of protons by the dye is a measure of the creation of Δψ, given the “voltmeter‐type” characteristics of the dye and its sensitivity to local fields. Treatment with bedaquiline and its binding to the c‐subunit in wild‐type membranes does not interfere with the continued succinate translocation from outside to inside (or with K^+^ translocation in the opposite direction) through the aqueous access channels in F_O_ and therefore with the local Δψ created by such translocation of succinate/K^+^. Capture of H^+^ by bedaquiline does not lead to collapse of the Δψ and hence no reversal of the fluorescence of Oxonol VI is observed in wild‐type IMVs in the data.^21^ However, incubation with 2 μM valinomycin in wild‐type membranes causes the valinomycin to perform its function as a carrier for K^+^ ions which are present at a 100 mM concentration in the outside medium. Thus K^+^ ions are translocated from outside to inside by valinomycin through the same aqueous channels in the membrane‐bound F_O_ portion of ATP synthase which has the right polarity to dissipate the Δψ created by succinate movement from outside to inside. This collapse of Δψ by valinomycin‐induced K^+^ translocation leads to rapid, almost complete reversal of the fluorescence in wild‐type membranes, as observed.

In mutant membranes, no reversal of fluorescence quenching of Oxonol VI by bedaquiline treatment can occur given that the antibiotic does not have the ability to bind to its c‐subunit site in the mutant and cannot influence Δψ events involving the anion/countercation. Hence bedaquiline has no effect on the magnitude of the local Δψ, as observed in both IMVs and whole cells. As the a–c interface is disrupted in the D32V mutant, the lack of significant effect of valinomycin‐K^+^ in the reversal of Oxonol VI fluorescence quenching is also explained, given that K^+^ needs to translocate through the same interface to exert its effects. However, the model for uncoupling given by Hards et al,^21^ which incorporates a general movement of K^+^ through the membrane cannot explain the reversal of fluorescence in wild type but lack of reversal in the mutant by 2 μM valinomycin in a 100 mM K^+^ medium because the same effect should have been obtained in both wild type and mutant according to their model. The authors of the study are aware of these difficulties and concluded that their results “suggest that the charge across the membrane is maintained through as yet undetermined processes during bedaquiline challenge.” These “undetermined processes” are highlighted by the two‐ion theory of energy coupling. Further, the dual role of succinate as an oxidation substrate and as a permeant ion is crucial to explain the lack of effect of bedaquiline treatment on Δψ observed in both IMVs and whole cells. It would have been interesting to know the results of experiments in the assay for measurement of Δψ on the reversal of Oxonol VI fluorescence by CCCP treatment in both wild‐type and mutant membranes. However, such experiments were not performed,^21^ and the author believes it would be valuable to design these experiments and collect such data in future work. This would lead to a better understanding of the bactericidal effects of bedaquiline due to its uncoupling action which are distinct from its delayed onset of killing arising from the effect of ATP depletion. It would also be useful to design experiments to unequivocally distinguish between bedaquiline's function as an *uncoupler* or as an *inhibitor*. These experiments may also help in optimizing the dosage of bedaquiline to be administered to patients of MDR‐TB during drug therapy.

It is proposed here that a novel, chemically attractive explanation and resolution of the above crucial issues lies in the fact that under the experimental conditions of Hards et al^21^ and Lamprecht et al^36^ that employ an 80‐fold or a 30‐fold to 300‐fold higher concentration of bedaquiline respectively, relative to its minimum inhibitory concentration, an H^+^/K^+^ antiport *ionophore* mechanism of bedaquiline and an uncoupling mode of action of the drug discussed above is operative, in accord with the central tenets of Nath's torsional mechanism in ATP synthase and two‐ion theory of energy coupling/uncoupling.^12^, ^13^, ^28^, ^37^ The collapse of the H^+^ and K^+^ electrochemical gradients induced by the H^+^/K^+^ exchange function of bedaquiline and the resulting uncoupling of ATP synthesis is responsible for the observed stimulation of oxygen consumption in *M. smegmatis* 21 and *M. tuberculosis*.^36^

**Figure 2:**
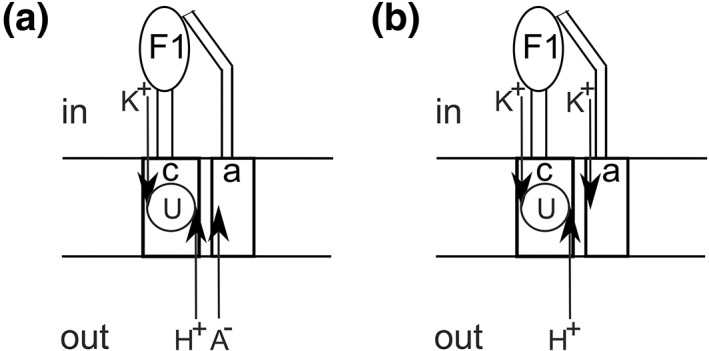
Model for the uncoupling action of bedaquiline and mycobacterial cell death resulting from subsequent metabolic consequences. The vast majority of ATP in a neutrophilic mycobacterial cell is produced by OXPHOS. Due to its high hydrophobicity, bedaquiline (shown here as U) binds from the outside bulk aqueous medium to its binding site on the c‐subunit at the rotor–stator a–c interface in the membrane‐bound F_O_ portion of the F_1_F_O_‐ATP synthase. Bedaquiline (U) captures protons translocated from the periplasm along their concentration gradient from the vicinity of their Glu/Asp‐61 (*E. coli* numbering) binding site on the c‐subunit, chelates K^+^ ions translocated from the cytoplasm along their concentration gradient, and mediates an electroneutral H^+^/K^+^ exchange, depicted by the circle in the schematic. The activity of bedaquiline as an H^+^/K^+^ ionophore releases H^+^ ions into the inside bulk aqueous medium, and K^+^ ions into the outside space, thereby dissipating the ΔpH and the ΔpK and creating a futile cycle of the ions that is uncoupled from ATP synthesis. As a result of the uncoupling of ATP synthesis caused by such ionophoric action of bedaquiline as an H^+^/K^+^ antiporter, respiration will be accelerated compared to the physiological case in the absence of bedaquiline, when protons and physiological succinate counteranions A^−^/K^+^ countercations translocate through F_O_, bind/unbind to/from their respective binding sites, and induce conformational changes in the c‐subunits of the c‐ring by means of ion‐protein interactions.^12^ However, bedaquiline does not interfere with the Δψ created by succinate translocation, A^−^ (A) or K^+^ translocation in the opposite direction (B) to their respective binding sites on the a‐subunit at the a–c interface in F_O_. Hence, the Δψ is not dissipated by bedaquiline, in contrast to the uncoupling induced by the classical anionic uncouplers of OXPHOS, such as the dinitrophenols, that dissipate both ΔpH and Δψ. In the model, the maintained Δψ in mycobacteria is postulated to be ultimately responsible for death of the organism by adversely impacting succinate homeostasis due to its continual translocation outwards coupled with protons by the redox reactions but blocked translocation inwards through the F_O_ portion of ATP synthase, thereby causing a depletion of internal succinate required for the physiological functioning of the Krebs cycle and the redox‐side of OXPHOS in cells. For further details, please refer to the text. The case of the combined operation of (a) and (B) has already been discussed and quantitatively analyzed during the conception and detailed formulation of Nath's torsional mechanism of energy transduction and ATP synthesis (e.g., in Reference 37, see especially table 2 of section 3.4 and eqs. 1–9)^37^

A schematic for the overall model for uncoupling according to the two‐ion theory of energy coupling is shown in Figure 2. It should be noted that translocation of K^+^ is *electrically* indistinguishable from succinate monoanion translocation in the opposite direction, and both possibilities are shown in Figure 2. However, the dissipation of *both* ΔpH and Δψ by the neutral, undissociated form of the classical anionic OXPHOS uncouplers is naturally explained by the lipophilic properties of the anionic uncouplers; a corresponding explanation in the case of K^+^ countertransport is somewhat more problematic. Regardless, the ability of bedaquiline to dissipate both the ΔpH and the ΔpK due to its activity as a genuine H^+^/K^+^ antiporter, but *not* the Δψ arising from succinate translocation (Figure 2A) or, equivalently, from K^+^ uniport translocation in the opposite direction to succinate translocation (Figure 2B) should be emphasized. The above difference has major biological implications that are discussed in the next section.

Before discussing the biological implications that arise, the inadequacy of other proposed explanations in the literature in this context and their inconsistency with experimental data ought to be stressed. Previously, Feng et al^38^ have proposed that bedaquiline exerts its effects by functioning as a cationic protonophore. However, the proposal does not explain how bedaquiline challenge dissipates only the ΔpH but not the Δψ in IMVs of *M. smegmatis*. Another proposal postulates that perturbation of the a–c interface leads to an uncontrolled proton leak uncoupled from ATP synthesis.^21^ However, the proposal fails to satisfy, at the same time, the twin conditions of: (a) dissipation of the transmembrane ΔpH and ΔpK/Δψ gradients that are required to cause uncoupling and enhancement of oxygen consumption rates, and (b) simultaneous maintenance of the Δψ by the proposed electroneutral process that couples the H^+^ leak with a general exchange of K^+^ ions through the membrane. Moreover, it should be understood that mere uncoupling and futile cycling of H^+^ and K^+^ in the cell arising from an ionophoric mode of action of bedaquiline is not sufficient by itself to explain the killing of mycobacteria by the drug.

## 7 MAJOR BIOLOGICAL IMPLICATIONS

The above has major biological implications. The key similarities and differences between the action of bedaquiline as uncoupler and the classical anionic uncouplers of OXPHOS, such as the dintrophenols can now be summarized. Like the classical uncouplers that dissipate both ΔpH and Δψ across the F_1_F_O_‐ATP synthase, the ionophoric properties of bedaquiline that cause it to function as an *H* ^*+*^ */K* ^*+*^ *exchanger* (Figure 2) lead to an “uncoupler‐like” mode of action upon bedaquiline challenge that dissipates the transmembrane H^+^ and K^+^ concentration gradients. However, the Δψ arising from succinate translocation (Figure 2A) is left virtually intact at its original value. In other words, bedaquiline treatment does not have the power to dissipate the Δψ in whole cells or IMVs. This key difference may lie at the heart of the bactericidal mode of action of bedaquiline. This implies that, upon bedaquiline treatment (unlike due to the action of the classical anionic uncouplers of OXPHOS), a local field will continue to exist in the aqueous a–c access channels of F_O_ (negative polarity inside). This maintained field arises from the noncollapsible electrical potential due to succinate monoanion translocation from outside to inside, or what is electrically equivalent, from a translocation of K^+^ from inside to outside. It should be noted that events within the energy‐transducing membrane can be rapidly communicated to the bulk aqueous phases. In fact, since the local field discussed above is within the aqueous access channels *normal* to the plane of the membrane, a Δψ will be “measured” across the bulk aqueous phases by use of permeant ions or dyes/probes from the measured distribution ratio of the motive probe and calculation based on the Nernst equation.

What are the mechanistic consequences of the above features for lethality? It has generally been thought that dissipation of ΔpH and Δψ is lethal to mycobacteria.^39^ However, based on the above interpretations, the origin of lethality in mycobacteria may be attributed to the continued *presence* of the Δψ. Continued redox‐linked coupled proton and succinate translocation, will interfere with the homeostasis of succinate, causing its depletion inside and accumulation outside, ultimately leading to death of the mycobacteria.

A final note on the electrochemical measurement of the restoration of the ΔpH and Δψ/ΔpA by the redox enzymes during physiological functioning is in order. In response to electron transfer, a primary ion translocation (say of H^+^) will create a Δψ that can be destroyed by a dye or permeant ion (e.g., K^+^ in the presence of valinomycin) as probe, and one can claim that a Δψ exists. However, the “measured” Δψ would have anyhow been destroyed subsequently by the secondary translocation (say of succinate ions) across the membrane. In other words, the existence of Δψ was only *transient* and would have been replaced by a succinate concentration gradient, or ΔpA, and the Δψ would disappear. All current theories of energy coupling (except Nath's two‐ion theory) postulate that only a “single ion” (e.g., H^+^) is the generator of both ΔpH and Δψ. However, the new theory proposes that ΔpH and Δψ are created by two *independent* agents, that is, H^+^ and succinate/K^+^, respectively. Failure to consider the critical role of the “second ion” that is also involved in biological energy transduction along with protons leads to inconsistencies and incorrect interpretations and has been shown to be the fundamental reason underlying past and present difficulties.^28^

To illustrate the above concept further for the redox reactions in animal mitochondria in the physiological mode of operation, let us call the cytochrome bc1 Complex III‐produced driving force as ΔpH and the driving force generated by Complex I (reduced form of nicotinamide adenine dinucleotide (NADH) oxidoreductase) and Complex IV (cytochrome oxidase) as Δψ, with both components measured by permeant ion probes. Since succinate is a dicarboxylic acid, the former is associated with the translocation of succinate monoanions and protons and the latter with cotransport of succinate dianions and protons. In the physiological pH range, the neutral form of succinic acid is very small (<10–15%), and can be neglected in the present discussion. With change in environmental variables of the medium (e.g., pH), an increase in the fraction of the monoanionic form of the succinate will necessarily lead to a decrease in the fraction of the dianionic form and vice versa. Hence, as found in several experiments in bioenergetics and bacterial flagellar motility, it would always appear that the sum of ΔpH and Δψ components of the driving force remains constant, and that a decrease in one component of the driving force is compensated for by an increase in the other component and vice versa. This conservation in the total magnitude of the driving force and the compensation of one component by the other actually arise from the chemical ionization properties of the cotransported succinate species, which, being a dicarboxylic acid, exists in both monoanionic and dianionic forms, and both forms need to be cotransported along with protons on the redox side in order to maintain homeostasis during steady‐state operation.^40^ These fundamental new mechanistic insights urgently need to be incorporated into the organized body of our biological knowledge for future scientific and technological progress.

## 8 CONCLUSIONS

Based on the interpretation of recent biophysical fluorescence quenching data and other biochemical experiments by a novel two‐ion theory of energy coupling in ATP synthesis, it has been concluded that the anti‐TB drug bedaquiline fumarate exerts its bactericidal function due to metabolic effects that arise from and follow its uncoupling mode of action. However, on further examination, it has been found that the mechanism of uncoupling by bedaquiline shows key differences from that of the classical anionic uncouplers of OXPHOS, which have been postulated to lie at the heart of the mechanism of lethality in mycobacteria. The classical anionic OXPHOS uncouplers have been shown to collapse both the driving forces ΔpH and Δψ, while bedaquiline only collapses the ΔpH but leaves the Δψ intact. It has been concluded that the results make logical sense only if the two driving forces ΔpH and Δψ are created by two independent agents, as predicted by the two‐ion theory of energy coupling in ATP synthesis by the F_1_F_O_‐ATP synthase, and not solely by protons, as in current dogma. The critical role of succinate co‐anion or K^+^ countercation translocation in energy transduction and coupling, the crucial ionophoric function of bedaquiline in H^+^/K^+^ antiport, the potent and specific uncoupling that results from localization of the drug to its target c‐ring in F_O_, and the consequent stimulation of respiration has been highlighted. The results show how stereospecific drug–target interactions in F_O_ lead to an acceleration of cellular respiration and to lethality of mycobacteria arising from a perturbation in succinate homeostasis over time (Figure 2). New experiments that offer further insights into the uncoupling mechanism by bedaquiline have been suggested. The experimental information has been integrated into a consistent model of the uncoupling and mycobacterial cell death actions of bedaquiline. The major biological implications of these fundamental insights for the interdisciplinary fields of bioenergetics, bacterial motility, respiration, and the design of antimicrobials have been discussed.
